# *De Novo* Transcriptome Hybrid Assembly and Validation in the European Earwig (Dermaptera, *Forficula auricularia*)

**DOI:** 10.1371/journal.pone.0094098

**Published:** 2014-04-10

**Authors:** Anne C. Roulin, Min Wu, Samuel Pichon, Roberto Arbore, Simone Kühn-Bühlmann, Mathias Kölliker, Jean-Claude Walser

**Affiliations:** 1 Department of Environmental Sciences, Zoology and Evolution, University of Basel, Basel, Switzerland; 2 Genetic Diversity Centre (GDC), ETH Zürich, Zürich, Switzerland; Universidade de São Paulo, Brazil

## Abstract

**Background:**

The European earwig (*Forficula auricularia*) is an established system for studies of sexual selection, social interactions and the evolution of parental care. Despite its scientific interest, little knowledge exists about the species at the genomic level, limiting the scope of molecular studies and expression analyses of genes of interest. To overcome these limitations, we sequenced and validated the transcriptome of the European earwig.

**Methodology and Principal Findings:**

To obtain a comprehensive transcriptome, we sequenced mRNA from various tissues and developmental stages of female and male earwigs using Roche 454 pyrosequencing and Illumina HiSeq. The reads were *de novo* assembled independently and screened for possible microbial contamination and repeated elements. The remaining contigs were combined into a hybrid assembly and clustered to reduce redundancy. A comparison with the eukaryotic core gene dataset indicates that we sequenced a substantial part of the earwig transcriptome with a low level of fragmentation. In addition, a comparative analysis revealed that more than 8,800 contigs of the hybrid assembly show significant similarity to insect-specific proteins and those were assigned for Gene Ontology terms. Finally, we established a quantitative PCR test for expression stability using commonly used housekeeping genes and applied the method to five homologs of known sex-biased genes of the honeybee. The qPCR pilot study confirmed sex specific expression and also revealed significant expression differences between the brain and antenna tissue samples.

**Conclusions:**

By employing two different sequencing approaches and including samples obtained from different tissues, developmental stages, and sexes, we were able to assemble a comprehensive transcriptome of *F. auricularia*. The transcriptome presented here offers new opportunities to study the molecular bases and evolution of parental care and sociality in arthropods.

## Introduction

Earwigs are widely distributed geographically and are important in ecology and agriculture as predatory and detritivorous insects. Some species are invasive and have successfully colonized non-native grounds after anthropogenic dispersal and have become pests (reviewed in [1]). Most earwigs are cosmopolitan foragers feeding on plant material including pollen, fruits, and detritus, but they also represent important predators of other invertebrates and their eggs. As a consequence, numerous earwig species are studied for their role in agricultural food webs to improve their efficacy as a biocontrol for pests such as aphids and the fall armyworm, *Spodoptera frugiperda* [2], [3]. Earwigs form part of the Polyneoptera, an insect lineage still rather poorly resolved phylogenetically [4], and are a phylogenetically ancient insect order (the Dermaptera). The earliest earwig fossils date back to the Jurassic and lowermost Cretaceous (*i.e.* more than 200 Mya, [5]). The order is characterized by the conspicuous sexually dimorphic un-segmented cerci (“forceps”, [6]), a typically ground-living, often gregarious and nocturnal life-habit, and the ubiquitous occurrence of forms of maternal care [1]. The order comprises approximately 1,800 species that are consistently organized in 11 families [7]. While the major phylogenetic position and structure of the order are now roughly established [7], [8], the details of the phylogenetic relationships among earwig species have not been fully resolved, partly due to lack of genomic data.

The European earwig (*Forficula auricularia*) is probably the most common and widely distributed earwig species in Europe. Native to the western Euroasian region, it was introduced by human activity in Northern America, Australia and New Zealand where it quickly established and is sometimes regarded as an invasive species and a pest in gardens and agricultural settings [1]. The European earwig is also the scientifically best-studied earwig species and has been used as experimental system in various evolutionary contexts, including sexual selection and the evolution of reproductive tactics, maternal care and family interactions [9]–[11]. Females show pronounced maternal care; they protect and clean the eggs, and they provide food and protection to hatched nymphs. While maternal care for the eggs is mandatory, it is facultative for later life stages since the nymphs are mobile and can survive without maternal care by self-foraging (reviewed in [1]). These conditions are thought to approximate ancestral conditions under which parental care originally evolved. Therefore, the European earwig (and other earwig species like *Anisolabis maritima* and *Euborellia annulipes*) is increasingly used as an experimental system to study the evolutionary origin and genetics of parental care and social behavior.

Yet, despite the scientific interest in earwigs, only little knowledge and data are available at the genomic or proteomic level. The first transcriptomic data of the European earwig was recently published in an attempt to improve the polyneopteran phylogeny [8]. Even though this transcriptome is a first step in the establishment of genomic/transcriptomic resources to study earwig biology in molecular terms, it was based on RNA extracted from only adult stage and yielded fragmented and incomplete sequence data. Thus, towards the improvement of the genomic resources needed to study for example gene or genome evolution, gene expression, or insect systematics, we aimed to establish a more comprehensive transcriptome of the European earwig. Here, we present and validate the draft transcriptome based on a hybrid assembly of Roche 454 and Illumina HiSeq data. In order to obtain a more exhaustive representation of transcripts, we combined different tissues (heads, thoraxes, abdomens, brain, and antenna) and developmental stages (eggs, nymphs and adults) from both males and females. As our analysis showed that the published transcriptome is fragmented, incomplete and lacking quality information, we deliberately did not use these published data for our hybrid assembly. After the assembly, we screened our transcriptome for putative microbial contamination. We also annotated transposable elements and removed redundancy, keeping alternative-splice variants. We then estimated the completeness and the fragmentation of our dataset by applying the core Eukaryotic gene mapping approach (CEGMA, [12]). Our transcriptome was also compared against other insect protein databases to determined protein-coding genes shared with eu-social and non-social insects. This sub-sample was annotated using Gene-Ontology (GO). We eventually established and validated qPCR by studying expression differences in males and females for 5 genes reported as being sex-biased in the honey bee [13]. We could confirm that some of these genes show expression differences between males and females but also between brain and antenna tissue in earwig. This method will allow us to study the expression of candidate genes putatively involved in maternal care and social behavior in the future. Further information on the assembly and links can be found at http://evolution.unibas.ch/walser/dermaptera.htm.

## Results and Discussion

**Figure 1:**
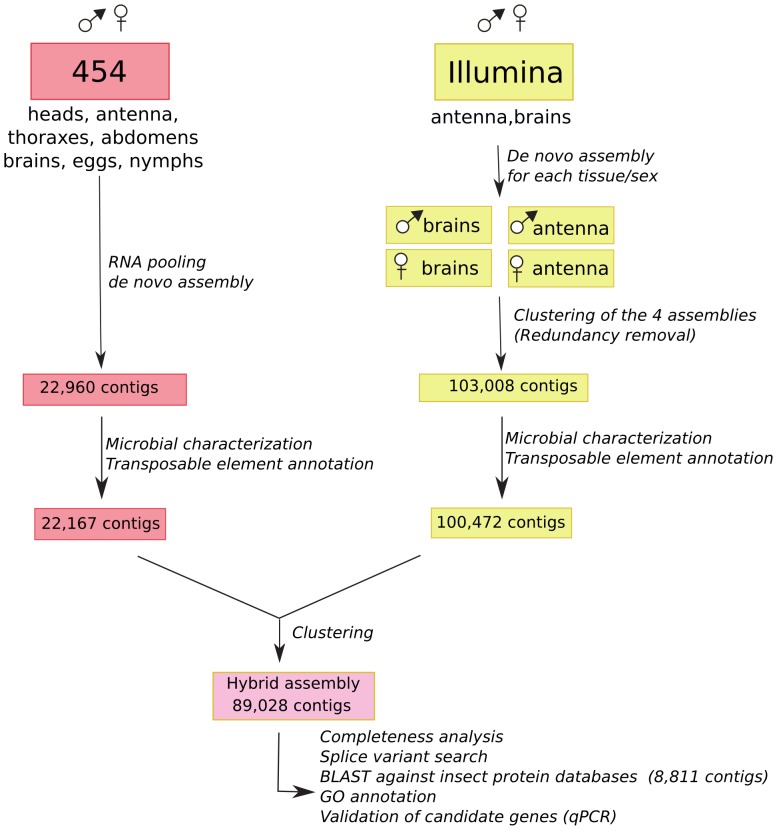
Flow chart of the hybrid assembly process.

A recent study showed that higher quality assemblies could be obtained when 454 and Illumina contigs are combined [14]. Following these guidelines, the Illumina and 454 reads were independently pre-assembled make use of an optimized *de novo* assembler platform. The initial Illumina and Roche 454 pre-assemblies (Fig. 1) resulted in 103,008 and 22,960 high quality contigs, respectively. The not assembled reads from the Roche 454 run, called singletons, were adapter trimmed, quality, and size selected but not included for further analysis. In a first step, the contigs were screened for possible contaminants and transposable elements. The remaining contigs were combined in a hybrid assembly resulting in 89,028 unique contigs.

### Characterization of non-earwig and transposable element sequences in the pre-assemblies

#### Microbiota screening

Earwigs, as many other organisms, live in close contact to microbial communities. Thus, we carefully prepared the samples in order to reduce level of possible contaminants (see Materials and Methods). In addition, the library preparation discriminated against non-polyadenylated molecules (poly-A enrichment, see Materials and Methods) and further reduced potential bacterial contaminants. Both steps reduced but did not entirely remove microbial contamination. To assess the level of potential remaining contaminants, we applied Pauda [15] to align the two pre-assemblies against a database of 56 million known proteins from Alveolata, Amoebozoa, Archaea, Bacteria, Fungi, Nematoda, Platyhelminthes and Viruses (Table S1).

**Figure 2:**
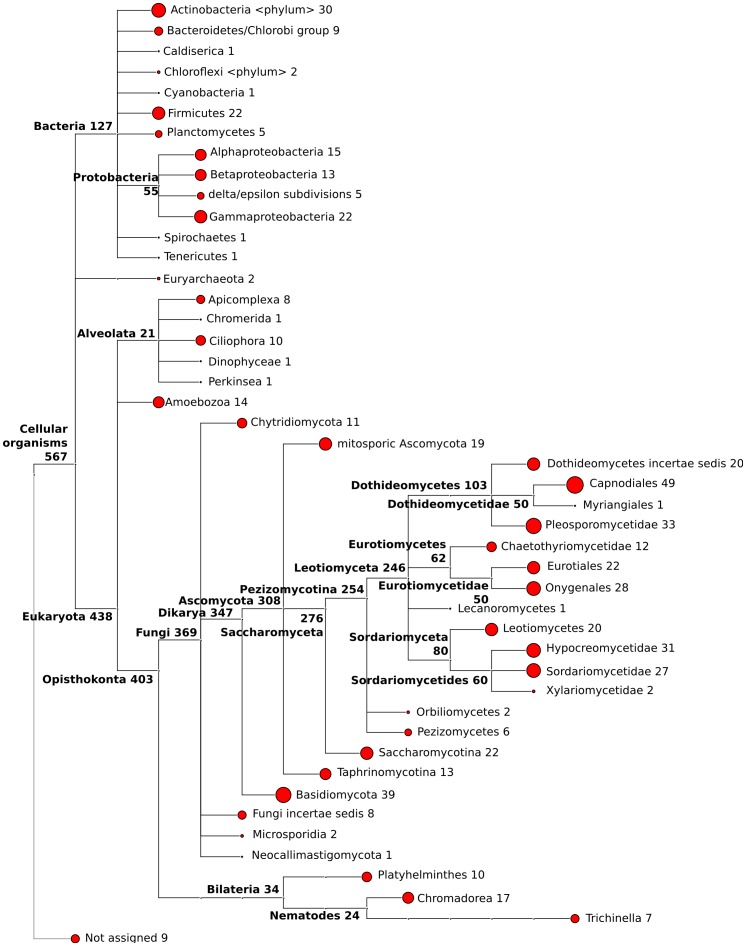
Taxonomic assignments of microbial contaminants using MEGAN. Assignment of all microbial SSU-LSU rRNA and mRNA sequences to the least common ancestor of their blastn and blastx hits, respectively. The red circle size are proportional to the number of sequences assigned to that node (maximum 49 reads), whereas the numbers are the accumulative sum of sequences assigned within subclades.

In total, 468 sequences (*i.e.* about 0.5% of all contigs) were putative homologs of microbial proteins. In addition, we identified 152 contigs corresponding to the small (SSU: 16S or 18S rRNA) or large ribosomal subunit (LSU: 23S or 28S rRNA), including 21 contigs specific to arthropods and therefore putatively of earwig origin (Table S1). Overall, we could assign about 23% of those contigs to a bacterial origin and 60% to a fungal origin (Fig. 2, Fig. S1 and Table S1). Out of the 50 top genera identified, 39 corresponded to fungi, 4 to bacteria and 1 amoeba all commonly found in soil samples. Interestingly, one of the identified fungi species is an already known parasite isolated from the habitat of the European earwig [16]. With this screening, it is likely that we identified part of the native microbiota of the earwig. Those sequences were removed from the pre-assemblies.

#### Transposable element screening

**Figure 3:**
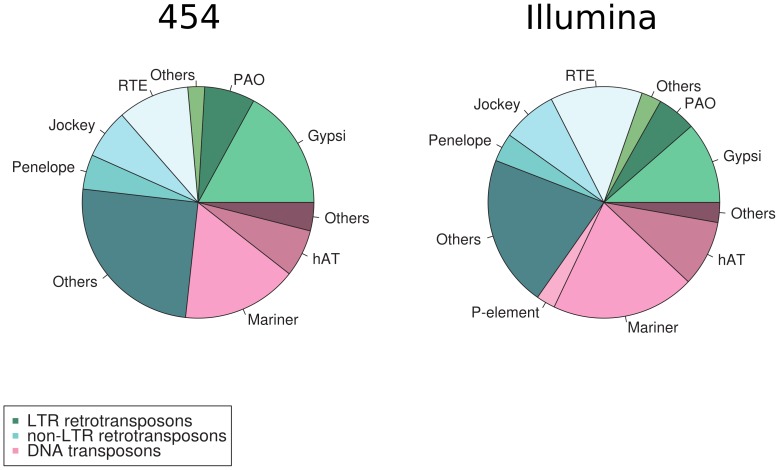
Most common transposable element distribution in the 454 and Illumina pre-assemblies.

Numerous studies documented that transposable elements (TEs) are pervasive and often constitute a substantial component of the size of a genome [17]. An unknown proportion of full-length TEs are transcriptionally active (*i.e.* transcribed) in a given genome at a given time [18]. Our approach does not discriminate against all TEs especially the retrotransposons which are polyadenylated [19]. Therefore, active TEs could inflate the number of contigs found in our assemblies and need to be identified and excluded from the final transcriptome. Therefore, we screened our preliminary assemblies for TE specific proteins using RepeatMasker [20]. We identified 2,076 and 694 contigs with significant similarity to known TE protein (Fig. 3 and Table S2). The fraction of retrotransposons (class I) and DNA transposons (class II) identified is similar to other transcripome studies in insects (e.g. [21]). In particular, Mariner and Gypsy elements seem to be common in the earwig transcripome. This finding is in agreement with previous work, which described the ubiquitous presence of these elements in insects [22]–[25] including earwigs [26].

### Completeness of the hybrid assembly

The 454 and Illumina pre-assemblies cleaned of microbial and transposable element sequences were combined and clustered to result in a hybrid assembly comprising 89,028 contigs (Fig. 1). To estimate the completeness of the hybrid assembly (hereafter designated as the earwig transcriptome), we compared the 89,028 contigs to a set of highly conserved and reliable annotated core proteins (n = 458) of *Drosophila melanogaster* and *Aedes aegypti* [12]. The Core Eukaryotic Genes Mapping Approach (CEGMA) showed that the 458 proteins of the core dataset could be unambiguously identified in our transcriptome, with a median value of completeness of 97%. Among those, 252 proteins were fully present (completeness >95%, Table S3). In order to put this into prospective, the previously published earwig transcriptome used for phylogenetic analysis (Simon et al. 2012) harbors a median value of completeness of 30%, with 20 full proteins only (Table S3). This comparison shows that our dataset contains a larger and/or less fragmented fraction of the earwig transcriptome. For this reason, the published transcriptome was not included in our hybrid assembly. This interpretation is also supported when comparing the CEGMA analysis of our transcriptome with the one from other published *de novo* transcriptome assemblies [27], [28].

### Identification and annotation of the earwig protein core set

Based on comparison with other insect species and the observation that gene number and average gene length are highly conserved among eukaryotes [27], we assume that approximately 200 Mb of the *F. auricularia* genome is organized in exons. Although we carefully removed potential microbial contamination, diminished TEs sequences, and even reduced redundant transcripts (see Materials and Methods), we believe that our dataset overestimates the number of protein coding genes, a common problem of RNAseq based transcriptome studies. The high number of contigs might also indicate the presence of non-coding transcripts (nc-RNA [29]), pseudogenes [30] or sequences errors (e.g. chimeras, [31]). It is also likely that a less stringent clustering could have reduced the number of contigs but also removed potential splice variants. In fact, we found evidence of putative variable transcripts. For example, we found two possible isoforms of the *RhoGAP-like* gene (Text S1). The mapping of the Illumina short reads using both isoforms as a reference supports this idea. Even though these preliminary results would need to be confirmed by qPCR, it indicates that one of the variants is more abundant than the other in the brain sample (data not shown).

**Figure 4:**
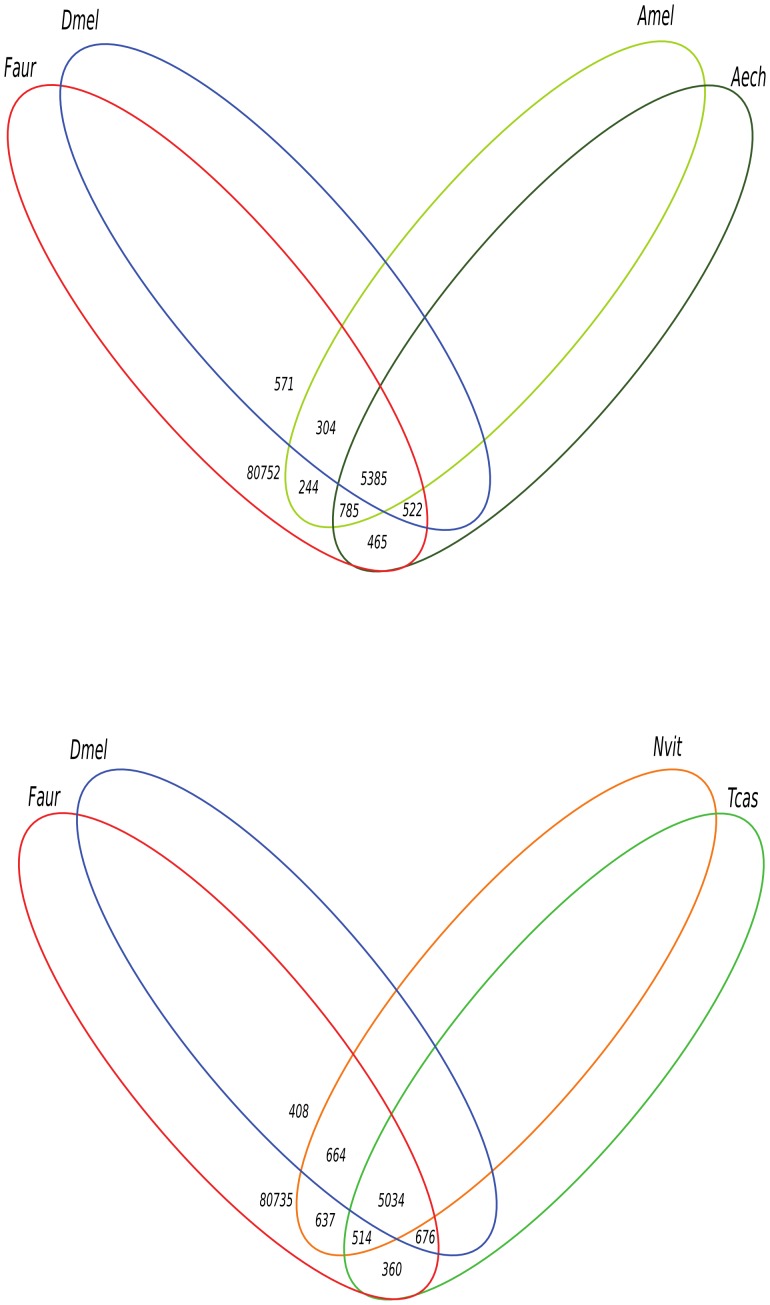
Venn-diagram of protein sequences shared by *F. auricularia* and 5 insect species. Numbers represent the number of proteins specifically shared by the particular combination of species. A) between *F. auricularia, D. melanogaster* and the social insects *A. mellifera* and *A. echinatior* B) between *F. auricularia, D. melanogaster* and the non-social insects *N. vitripennis* and *T. castaneum*.

A BLAST search using our contigs as query against 2 social and 3 non-social insect databases *i.e Apis mellifera* (honey bee), *Acromyrmex echinatior* (leaf-cutting ant), *Drosophila melanogaster* (fruit fly), *Tribolium castaneum* (red flour beetle) and *Nasonia vitripennis* (jewel wasp) revealed 8,811 contigs shared between our transcriptome and a least one of the five reference insect genomes (Fig. 4). Among those, only 2,400 could be found in the previous published transcriptome [8], which further confirms the completeness of our hybrid assembly. The completeness analysis was performed again using the 8,811 identified contigs. The same results as with the whole transcriptome were obtained (458 proteins identified, completeness of 97%), suggesting that these contigs, even though not representative of the whole transcriptome, constitute the earwig core protein dataset.

This subset of 8,811 contigs was then assigned for Gene Ontology terms (GO; [32]) using Blast2GO and based on blastx hits against the Swiss-Prot database. We were able to assign the contigs to the following categories (in terms of their numbers): molecular function: 1,046; cellular component: 2,021; biological process: 7,018 (Fig. S2). Altogether, the binding proteins and catalytic activity represent the vast majority of the molecular function category. Most of the contigs associated with the cellular component were assigned to the cell and the organelle part while those associated with a biological process were mainly involved in the cellular and metabolic process. Although GO term annotations are more relevant in the context of comparative analysis (between developmental stages for example), these results are congruent with findings in other insect transcriptome studies [33], [34] and confirm that we obtained the sequences of genes involved in central pathways. This was further illustrated by the KEGG metabolic pathways analysis (see Table S4), which allowed us to identify pathways involved for example in the purine (189 genes), pyrimidine (76 genes), or inositol-phosphate (45 genes) metabolisms.

Our comparative analysis also indicates that 124 (1.4%) of the identified 8,811 contigs might be specific to social insects (e.g. *A. mellifera* and *A. echinatio*r, *F. auricularia*) and absent from non-social insects (e.g. *D. melanogaster, T. castaneum* and *N. vitripennis*). 75 transcripts could be assigned to a molecular function, the most prevalent categories being protein-binding (52 transcripts) and proteins associated with a catalytic activity (23 transcripts, data not shown). These 124 contigs constitute possible candidates to further investigate the genetic bases of maternal care and extended social behavior (*i.e.* caste determination and task-specialization).

### Validation of the transcriptome and candidate gene expression analysis

**Figure 5:**
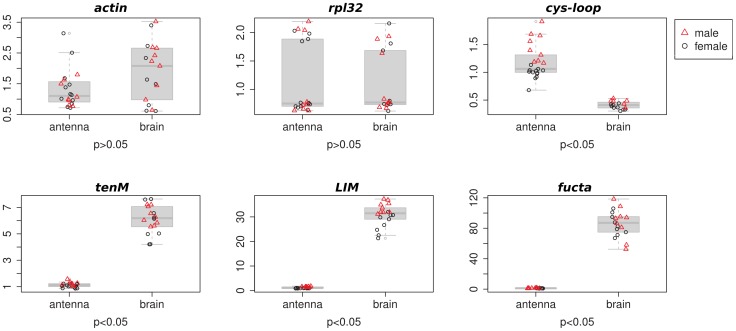
Gene expression for 2 housekeeping genes (*actin* and *rpl32*) and 4 candidate genes. Red triangles display male samples. Black circles display female samples. P-values indicate whether the gene is significantly differentially expressed between brain and antenna samples. * display genes which harbor a sex-biased expression.

We selected 7 housekeeping genes (*actin, EF1, mnf, rpl32, rpl20, tubulin* and *18S*) used as qPCR internal standards in *Drospohila melanogaster* [35]. Five of the selected housekeeping genes *(actin, EF1, mnf, rpl32* and *tubulin*) showed homologous sequences in our transcriptome and four of them (a*ctin, EF1, mnf* and *rpl32*) could be successfully amplified with earwig specific primers (Table S5). Using primers specific for the *18S* from *D. melanogaster* [36], we also successfully amplified this gene in our earwig samples. Yet, the stability test (see Materials and Methods) indicated that the *EF1* and *18S* genes could not be used as potential standards. In addition, because *mnf* showed significant sex-biased expression in both brain and antenna (wilcoxon test p<0.5, Table S6), the a*ctin* and r*pl32* genes were the only standards kept for further analysis (Fig. 5).

We also selected 5 candidate genes (c*ys-loop, NAD-like, LIM, tenM* and *fucta*) for which sex biased expression has been reported in the honey bee, *A. mellifera* [13], and compared their expression level between sexes (adult males versus females) and tissues (antennae versus brain). *NAD-like* was excluded from further analysis because most of the *NAD-like* samples did not meet the Ct8 criteria (See Materials and Methods). However, we confirmed sex-biased expression for the *cys-loop* and *LIM* genes in our system (Fig. 5, Table S6). In addition, significant expression differences between brain and antenna samples could be observed for the 4 selected genes (Fig. 5, Table S6). Interestingly, the c*ys-loop* gene showed higher expression in antenna than in brain. This gene has been described as a ligand-gated ion channel, *i.e.* a receptor that converts chemical signals to electrical signals. It is therefore not surprising to observe such an expression pattern between the olfactory tissue antenna [36] and the central system (brain). These results demonstrate that our transcriptome can further be used to develop gene primers and to study candidate gene expression. The established qPCR approach presented here will allow and thus enhance the study of the molecular evolution of social behavior in our system.

### Database

In order to facilitate the search of sequences of interest, we provide a searchable database at http://evolution.unibas.ch/walser/dermaptera.htm. This database allows to perform BLAST searches separately on the different data-sets described in the manuscript, *i.e.* the complete hybrid assembly (n = 89,000 contigs), core earwig proteins (n = 8,811 contigs), transposable elements (n = 2,076), microbitoa (N = 620), unassembled 454 reads singletons (n = 124,630).

## Conclusion

The European earwig, *Forficula auricularia*, is an organism studied in evolutionary, ecological and agricultural research. It is an important and very interesting insect system for the study of the evolution of reproductive tactics [9], and the early evolution of parental care and family interactions [37]. Despite the broad interest in earwigs, only limited and incomplete data existed at the molecular level. In this study, we showed that our transcriptome provides a substantial portion of the genes present in the European earwig, which is an important first step to enhance our ability to investigate the genetics and genomics of this species as well as other Dermaptera and insects.

## Materials and Methods

### Ethics statement

No specific permits were required for the described experiments. The European earwig is not an endangered or protected species.

### Earwig sample

The earwigs used for this study were part of a breeding line that originated from the progeny of three earwig females caught in Dolcedo (region Liguria), Italy in July 2008. These females were among a group of six females and six males caught on two adjacent olive trees. The females probably had already mated upon capture, but to ensure mating, the six females were set-up jointly with six males in the laboratory for continued mating until oviposition. The offspring of the selected females were used to establish a laboratory breeding line (line FaDo-08i). For mating, the offspring were set-up in containers of about 120 individuals each (approximately 60 males and 60 females). For each subsequent generation offspring of 5–10 females were chosen to continue the line. At the time when the individuals were sampled for the current study on May 5–6^th^, 2011, the line had been kept for four (adult tissues) and five (eggs/juveniles tissues) generations, respectively. For more details about rearing conditions, see [38].

### RNA isolation and sequencing procedure

Male and female adult earwigs, eggs and whole nymphs from all five juvenile stages (eggs, juvenile instars L1–L4) were selected from the breeding line FaDo-08i for total RNA isolation. Prior to dissection, the animals were exposed to petroleum ether (Sigma-Aldrich #77379) vapor. The digestive tract was carefully removed from adult animals to minimize possible contamination from gut content and microbes. We collected whole heads, antenna, thoraxes, abdomens, and dissected brains of five adult females and five adult males. We further sampled about 15 oocytes from one female, collected 10 nymphs from the L1 and L2 developmental stages, and five nymphs from the L3 and L4 stages. All samples were stored in RNAlater (Qiagen), a RNA stabilizing reagent, after dissection. A TRIzol (Invitrogen) protocol was used to isolate total RNA. The Roche 454 run was split into two half plates and two libraries from pooled samples were prepared. Equal amounts of RNA from the whole heads and thoraxes of female and males were pooled for the first library. For the second library the abdomens of female and males, the oocytes, and the nymphs were combined in equivalent amounts. Approximately 2 μg of total RNA from the pooled samples was used for the cDNA library construction and subsequent sequencing. The library preparation and run was performed at the Functional Genomic Center in Zurich (For more details see Text S2). For the Illumina HiSeq run libraries for the brain and antenna tissues from females and males were prepared separately using Illumina TruSeq kit with index following the manufacture's protocol. The single read (SR) 100 nt and 150 nt multiplex HiSeq run was performed at the Quantitative Genomics Facility (QGF) in Basel.

### *De novo* pre-assemblies

The Roche 454 and the Illumina datasets were assembled separately. A detail schematic of the sample design and the different assembly steps are provided in Figure 1.

For the 454 data the quality filtering, the read trimming, and the transcriptome assembly were generated using GS De Novo Assembler (version 2.7; Roche, Switzerland). Because the unassembled reads (i.e. singletons) still contain the adaptor sequences, the reads were trimmed and size selected using cutadapt [39] version 1.2.

PRINSEQ lite [40] was used for quality assessment and filtering of the SR100 and SR150 Illumina reads prior to the de novo assembly performed with CLC Genomic Workbench (Version 6.0.1). The four individually assembled transcriptomes (e.g. female brain, male brain, female antenna, and male antenna) were concatenated and usearch (version 7.0, [41]) with a 95% identity clustering to reduce redundancy was applied.

### Contamination analysis

Initial 454 and Illumina contigs were submitted to Bowtie2 v2.1.0 [42] and Pauda v1.0.1 [15], where they were mapped to reference proteomes. These latter were downloaded as of May 2013 from the NCBI website (http://www.ncbi.nlm.nih.gov/Taxonomy/Browser/wwwtax.cgi, Accessed 2014 March 15) by independently selecting all proteins sequences from Amoebozoa (about 0.2 million of proteins), Alveolata (0.5 m), Archaea (1.7 m), Bacteria (46.5 m), Fungi (2.9 m), Platyhelminthes (0.1 m), Nematoda (0.3 m) and Viruses (2.2 m) (total of about 56.4 m). Briefly, individual contigs were translated using all six reading frames into proteins and fast aligned, using default parameters, to the above reference proteins. The blastx scores were parsed using local perl scripts and used to rank the microbiota. Only blastx results with an alignment length over 33 amino acids to the reference proteins, a similarity over 75% and e-value below 10^−10^ were considered as positive hits. Results were visualized in MEGAN v4.0.1 [43]. While inspecting the data we ignored reads unassigned to taxa. Sequencing reads were also submitted to the r115 database of ARB-SILVA (release date: August 2013, https://www.arb-silva.de/no_cache/download/archive/release_115/Exports/) [44] to a local blastn search to identify small (SSU: 16S and 18S) and large (LSU: 23S and 28S) subunits of ribosomal RNAs of Bacteria, Archaea and eukaryotic organisms. Only blastn hits with an alignment length over 100 nt to reference rRNA sequences, an identity over 75% and e-value below 10^−15^ were considered as positive SSU and LSU.

### Transposable element identification

Contigs from the 454 run and the combined Illumina data were screened for the presence of transposable elements using the protein based database search provided by RepeatMasker [20]. Contigs whose 90% of the total length showed homology with a TE protein were excluded from the hybrid assembly (see Fig. S3 for distribution). Singletons were deliberately not analyzed.

### Clustering and hybrid assembly

Possible redundancy of the combined contamination-reduced 454 contigs and Illumina dataset as well as the singletons was reduced using usearch (version 7.0, [41]) and CAP3 [45]. The hybrid assembly of the combined 454 contigs and the Illumina contigs resulted in a total of 89,028 sequences. The hybrid assembly together with the clustered singletons (deliberately not included for further analysis) builds the transcriptome of the European earwig. A BLAST server will be made available upon acceptance of the manuscript for publication. The parameters for the clustering were carefully determined in order to reduce redundancy without removing possible alternative transcripts. In order to identify putative splice-variants, contigs of the hybrid assembly were BLAST searched against the *D.melanogaster* Exon Database (http://proline.bic.nus.edu.sg/dedb/, Accessed 2014 March 15). Contig pairs showing homologous relationship with the same gene of *D. melanogaster* but with different exons and showing 100% of sequence identity with each other for a 300 bp region were considered as potential gene isoform.

### Completeness analysis

The completeness of the hybrid assembly and of the published transcriptome was determined by performing a tblastn search using our transcriptome contigs as query against the CEGMA core genes dataset of *D. melanogaster* and *A. Aegypti* (http://korflab.ucdavis.edu/datasets/cegma/, [12], Accessed 2014 March 15). Custom Perl scripts were used to assess the completeness of our transcritpome (% coverage between query and core protein alignments). Only local alignments with e-value<10^−6^ were taken into account. Only the best BLAST hit results were kept (allowing only 1 contig per protein) so that the completeness analysis also reflects the transcritpome fragmentation.

### Protein comparison with insect databases, GO term analysis

Contigs were used in a reciprocal best-hits BLAST approach [46] to find homologues with *Apis mellifera* (honeybee, [22], http://hymenopteragenome.org, Accessed 2014 March 15), *Acromyrmex echinatior* (leaf-cutter ant, [47], http://www.antgenomes.org, Accessed 2014 March 15), and *Drosophila melanogaster* (fruit fly, [48], ftp://ftp.flybase.net, Accessed 2014 March 15), *Tribolium castaneum* (flour beetle, [49], http://beetlebase.org/, Accessed 2014 March 15) and *Nasonia vitripennis* (parasitic wasp, [50], http://hymenopteragenome.org/nasonia/, Accessed 2014 March 15). BLAST hits with a score <50 and e-values > than 10^−6^ were not considered for further analysis.

Gene ontology (GO) annotation was performed using Blast2GO version 2.5.1 [32], using the NCBI Blast service and a cut-off value of 10e^−6^ for the blastx search against the Swiss-Prot database. Categories represented by more than 15 sequences were taken into account. Blast2GO was also used to identify the metabolic pathways based on the Kyoto Encyclopedia of Genes and Genome (KEGG; [51]) and the Swiss-Prot database.

### qPCR establishment and validation of candidate gene expression

Earwigs from the same breeding line as the ones used for Illumina sequencing (from the eighth generation since the line was established) were used to extract RNA from both male and female brains and antenna. The experiment consisted of 40 females and 40 males and the RNA was extracted from brains and antenna at the stage when females were guarding their clutch of eggs. As before, the insects were sacrificed before dissection by exposure to petroleum ether. The protocol of RNA extraction is the same as described above. In order to obtain sufficient amount of RNA for qPCR, the extracted RNA from 10 males or 10 females were pooled for each tissue resulting in 4 biological replicates per sex and tissue. The extracted RNA was treated with DNaseI (Fermentas) to remove genomic DNA, and quantified in Qubit 2.0 Flurometer with RNA BR (Broad-Range) Assay Kit (Invitrogen). The quality of the extracted RNA was then controlled with the 8-capillary *NanoDrop* 8000 (Thermo Scientific). The cDNA library was prepared using GoScript Reverse Transcription System (Promega). An intron control PCR was run to confirm that the RNA samples were free of genomic DNA. The 5× HOT FIREPol EvaGreen qPCR Mix Plus (ROX) were used for runs on Applied Biosystems 7500 Fast Real-Time PCR System.

5 candidate genes (c*ys-loop, NAD-like, LIM, tenM* and *fucta*), known to harbor sex-biased expression in honey bee (*A mellifera* [13], and showing homologous sequences in our transcriptome (Table S5) were chosen for the analysis. For internal control, we selected 7 commonly used housekeeping genes (a*ctin, EF1, mnf, rpl32, rpl20, tubulin* and *18S* [35]). Primers were designed to discriminate potential genomic DNA (Table S5). The amplification efficiency was calculated in LinRegPCR (11.4 [52]) and genes with an efficiency range between 1.8 to 2.0 were kept for further analyses. The expression stability of the housekeeping genes was tested in each RNA pool (brain and antenna in both male and female) using geNorm, which is implemented in qbase^PLUS^ [53]. The expression of candidate genes was calculated using 2ΔΔCt method [54]. For each of the 4 biological replicates, 3 technical replicates were used. Melting curves were used to control the quality of the PCR products. Samples that did not meet the Ct8 value criteria (*e.g.* difference between the no reverse transcriptase control and the tested sample values greater than 8) were excluded from further analysis. The significance of expression differences between male and female or brain and antenna samples were tested in R (v.2.14.1 [55]) with a Wilcoxon test.

## Supporting Information

Figure S1 **Pie-charts of microbial contaminant taxonomic assignments at the phylum, class and family level.**
(TIF)

Figure S2 **Gene ontology annotation (molecular function, cellular component and biological process) of the 8,811 contigs conserved among insects.**
(TIF)

Figure S3 **Distribution of the proportion of the protein masked by repeat masker.** Red bars show contigs which have been removed from the assembly, *e.g.* sequences for which 90% of the length is masked (TE sequences).
(TIF)

Table S1 **Counts of mRNAs encoding microbial proteins and rRNAs contaminants.** SSU/LSU counts in brackets drawn from 454 and Illumina contigs, and their taxonomy assignments. nd: not determined.
(XLS)

Table S2 **Distribution of transposable element related proteins identified in the 454 and Illumina pre-assemblies.**
(XLS)

Table S3 **Completeness analysis.** The table provides data for the Core eukaryotic genes dataset proteins identified in our transcriptome. The table describes the length of the CEGMA protein, the completeness (% of the sequence identified in our transcriptome) and 3 parameters (sequence identity, e-value, score) of the BLAST output.
(XLS)

Table S4 **KEGG analysis results.** The table provides the number of contigs of the earwig transcriptome involved in a given KEGG pathway.
(XLS)

Table S5 **qPCR candidate and housekeeping gene sequences and primers.**
(XLS)

Table S6 **Wilcoxon test results for biased expression in sex and tissues.**
(XLS)

Text S1 **Alignment of the two variant of the** ***RhoGAP-like*** **gene.**
(FASTA)

Text S2 **Sample and library preparation for Roche 454.**
(DOC)
