# Supplementary figures and images for: De Novo Transcriptome Hybrid Assembly and Validation in the European Earwig (Dermaptera, Forficula auricularia)
Source: PLoS One. 2014 Apr 10;9(4):e94098. doi: 10.1371/journal.pone.0094098 (PMC3983118; doi:10.1371/journal.pone.0094098)

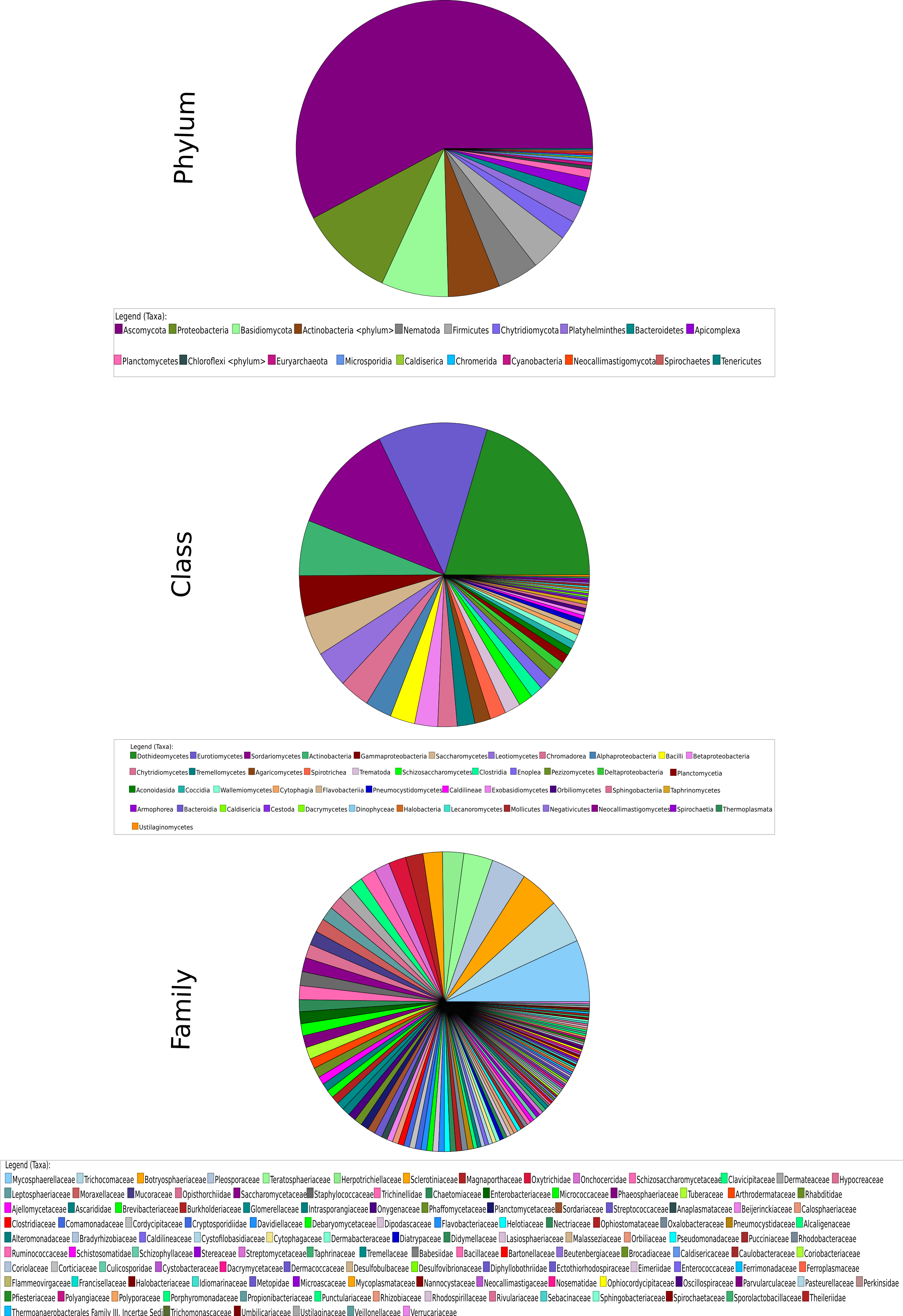

Supplement: Figure S1 — Pie-charts of microbial contaminant taxonomic assignments at the phylum, class and family level. (TIF) [file pone.0094098.s001.tif]

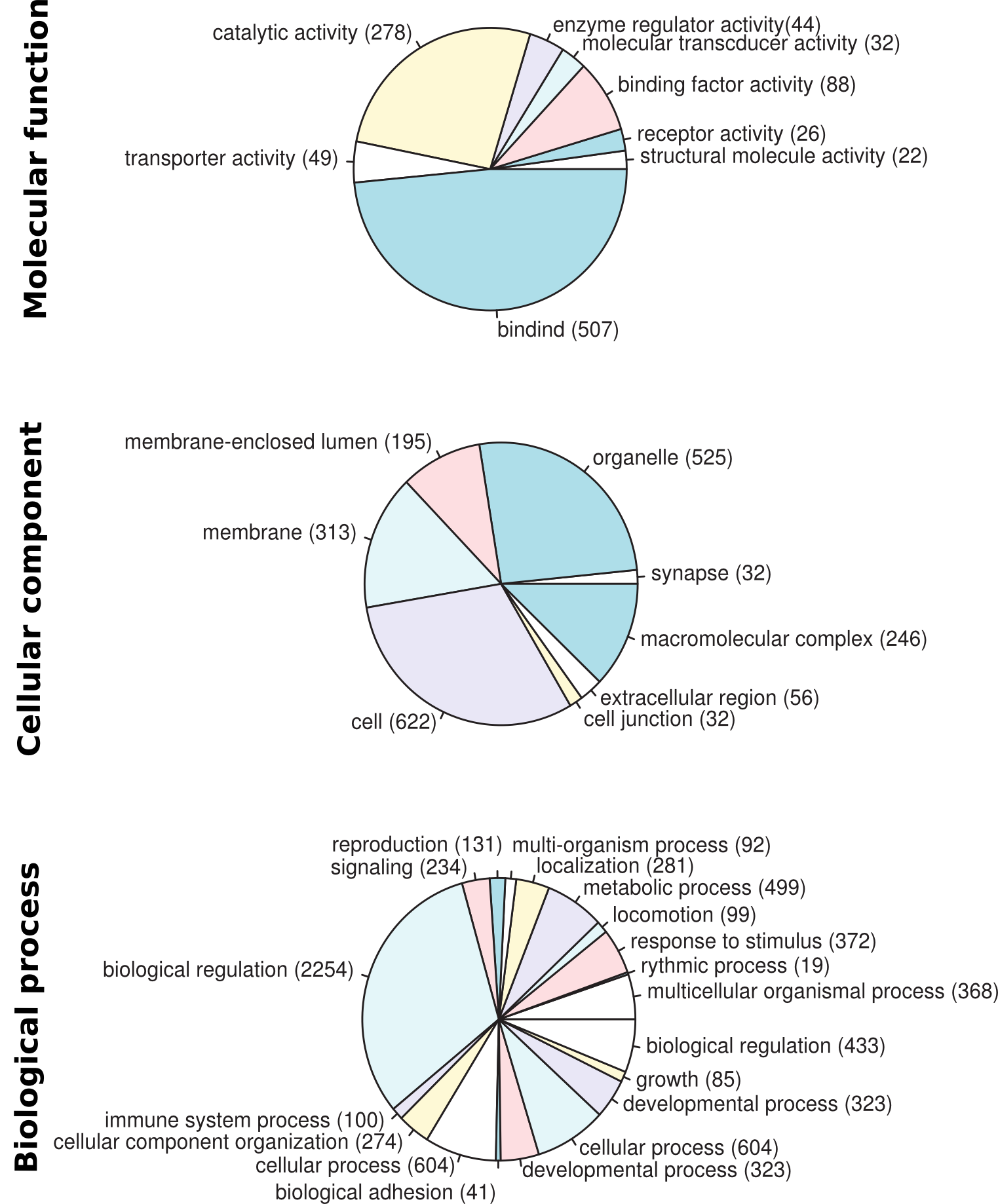

Supplement: Figure S2 — Gene ontology annotation (molecular function, cellular component and biological process) of the 8,811 contigs conserved among insects. (TIF) [file pone.0094098.s002.tif]

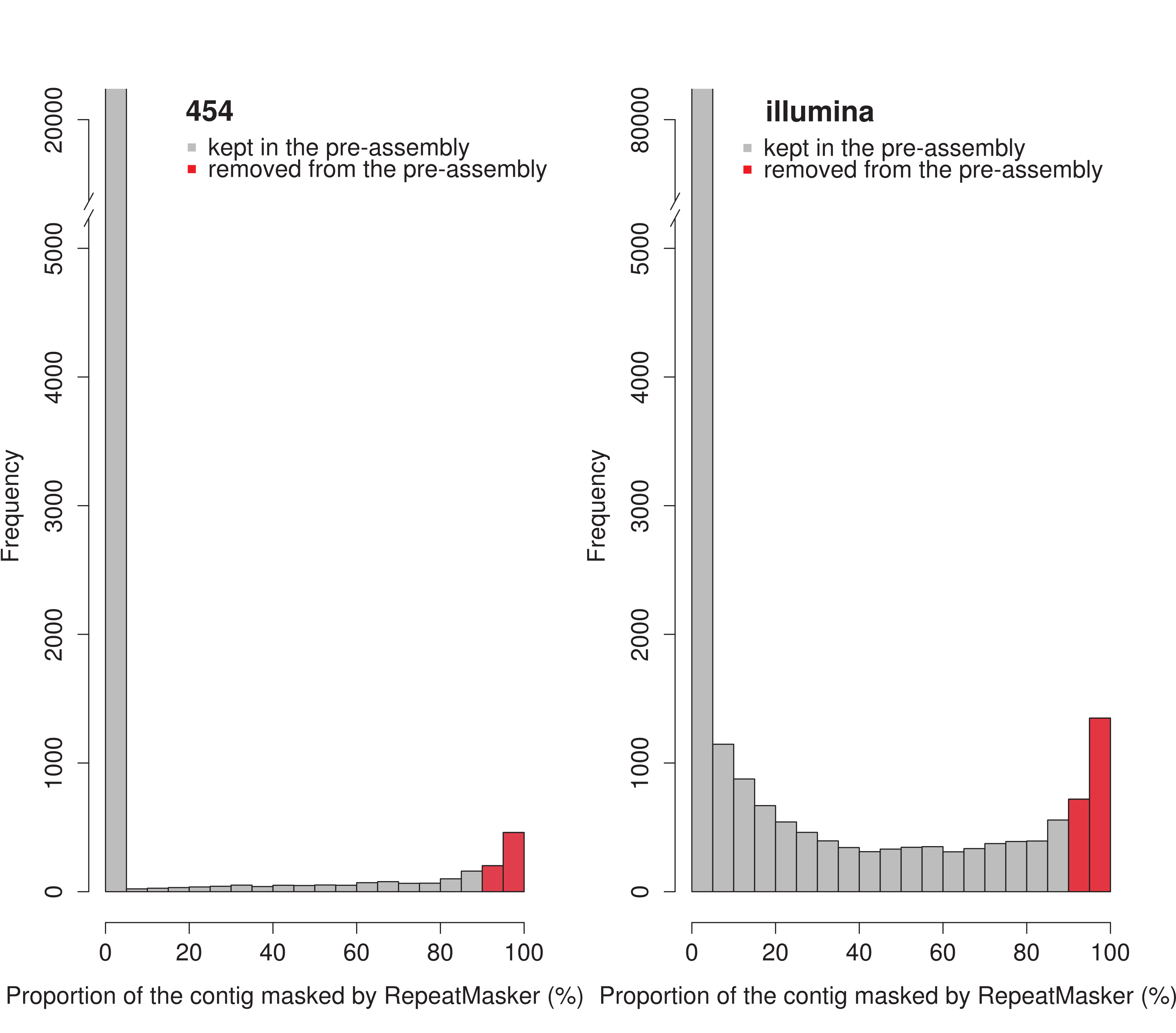

Supplement: Figure S3 — Distribution of the proportion of the protein masked by repeat masker. Red bars show contigs which have been removed from the assembly, e.g. sequences for which 90% of the length is masked (TE sequences). (TIF) [file pone.0094098.s003.tif]
