# Supplementary material for: De Novo Transcriptome Hybrid Assembly and Validation in the European Earwig (Dermaptera, Forficula auricularia)
Source: PLoS One. 2014 Apr 10;9(4):e94098. doi: 10.1371/journal.pone.0094098 (PMC3983118; doi:10.1371/journal.pone.0094098)
Supplement: Text S2 — Sample and library preparation for Roche 454. (DOC) [file pone.0094098.s011.doc]

**Sample and library preparation for Roche 454**

cDNA was synthesized from 2µg of total RNA for each sample using the MINT cDNA synthesis kit (Evrogen, SK001) following manufacturer instructions. The primers used in the MINT cDNA synthesis kit were substituted as follows: The 1µl of 3'primer (10µM) required in the kit was substituted by 1µl of PolTdeg (10µM, 5'-3': AAG CAG TGG TAT CAA CGC AGA GTA CTT TTG TTT TTT TTT CTT TTT TTT TTV N). Whereas the 2µl of the PCR Primer M1 (10µM) was substituted by 1µl of M1ACGG (10µM, 5'-3': AAG CAG TGG TAT CAA CGC AGA GTA CGG) and 1µl of polTM1 (10µM, 5'-3': AAG CAG TGG TAT CAA CGC AGA GTA CTT TTG TCT TTT GTT CTG TTT CTT TTV N). cDNA normalization results in equalization of the abundance of different transcripts and increase in the number of previously non-detectable genes in cDNA samples. Therefore normalization of the cDNA was performed using the TRIMMER cDNA Normalization kit (Evrogen, NK001) following manufacturer instructions. Approximately 500ng of normalized cDNA of each sample were used to generate a single strand cDNA (sst cDNA) transcriptome library on the Roche/454 Life Sciences GS-FLX Titanium platform (Roche, Basel, Switzerland) following the Rapid Library Preparation Method Manual. Briefly, the sst cDNA of each sample was nebulized and oligonucleotide adaptors were ligated to the fragments. One adaptor contained a barcode sequence that was used to discriminate the samples after sequencing as all libraries were combined in a single pool for further processing. Transcriptome library sequencing was then performed according to the Roche GS-FLX XLR70 Titanium emPCR and sequencing manuals. The pooled sample was sequenced on a full picotiterplate on a Genome Sequencer FLX Instrument. Sequencing raw data was processed with the GS Run Browser (version 2.5.3) using standard quality filtering and trimming as defined by the default settings. A total of 1,361,445 reads passed the initial filters with a median length of about 272nt.
